# One-Step Phytofabrication Method of Silver and Gold Nanoparticles Using *Haloxylon salicornicum* for Anticancer, Antimicrobial, and Antioxidant Activities

**DOI:** 10.3390/pharmaceutics15020529

**Published:** 2023-02-04

**Authors:** Reham Samir Hamida, Mohamed Abdelaal Ali, Haifa Essa Alfassam, Maha Abdullah Momenah, Mariam Abdulaziz Alkhateeb, Mashael Mohammed Bin-Meferij

**Affiliations:** 1 Laboratory for Nanobiology, Institute for Protein Research, Osaka University, Osaka 565-0871, Japan; 2 Plant Production Department, Arid Lands Cultivation Research Institute, City of Scientific Research and Technological Applications (SRTA-CITY) New Borg El-Arab, Alexandria 21934, Egypt; 3 Department of Biology, College of Science, Princess Nourah bint Abdulrahman University, Riyadh 11671, Saudi Arabia; 4 Histopathology Unit, Research Department, Health Sciences Research Center (HSRC), Princess Nourah bint Abdulrahman University, Riyadh 11671, Saudi Arabia

**Keywords:** plant-mediated synthesis, metallic nanoparticles, bacteria, colon cancers

## Abstract

Among various routes of metallic nanoparticle (NPs) fabrication, phytosynthesis has significant advantages over other conventional approaches. Plant-mediated synthesis of NPs is a fast, one-step, ecobenign, and inexpensive method with high scalability. Herein, silver (Ag) and gold (Au)-NPs were extracellularly synthesized using aqueous *Haloxylon salicornicum* (H@Ag-, H@Au-NPs) leaf extracts. GC-MS was performed to analyze the chemical compositions of *H. salicornicum* extract. H@Ag- and H@Au-NPs were characterized via UV-Vis spectroscopy, Fourier transform infrared spectroscopy, X-ray diffraction, transmission and scanning electron microscopy, and Zetasizer. H@Ag- and H@Au-NPs have surface plasmon resonance at 435.5 and 530.3 nm, respectively. FTIR and GC-MS data suggest that secondary plant metabolites and hydrocarbons might be responsible for the reduction and stabilization of NPs. XRD demonstrated that both NPs have a crystalline nature. H@Ag-NPs have a uniform spherical shape, whereas H@Au-NPs are spherical with few oval and triangular shapes, and their average nanosizes were 19.1 ± 0.8 and 8.1 ± 0.3 nm, respectively. Hydrodynamic diameters of H@Ag-NPs and H@Au-NPs were 184.7 nm, 56.4, and 295.4 nm, and their potential charges were −24.0 and −24.4 mV, respectively. The inhibitory activity of 500 µg/mL H@Ag- and H@Au-NPs was tested against Sw480, Sw620, HCT-116, and Caco-2 colon cancer cell lines and two normal cell lines, including HFs and Vero. H@Ag-NPs revealed potent anticancer activity against all cancer cells at low concentrations. Sw480 was the most sensitive cell to H@Ag-NPs, whereas Sw620 was the least permeable one. These findings suggested that the antiproliferative activity of H@Ag-NPs is cell-response-dependent and may be influenced by a variety of factors, including the cellular metabolic state, which influences cellular charge and interactions with charged NPs. Although H@Au-NPs were smaller, their reactivity against cancer cells was weak, suggesting that the chemical properties, metal structure, quantity and chemistry of the functional groups on the NP surface may influence their reactivity. The biocidal activity of 1 mg/mL H@Ag- and H@Au-NPs against *Staphylococcus aureus*, *Bacillus cereus*, *Escherichia coli* and *Klebsiella pneumoniae* was assessed. H@Ag-NPs showed biocidal activity against Gram-positive bacteria compared to Gram-negative bacteria, whereas H@Au-NPs showed no inhibitory activity. FRAP and DPPH assays were used to determine the scavenging activity of the plant extracts and both NPs. H@Ag-NPs (1 mg/mL) had the greatest scavenging activity compared to tested drugs. These findings suggest that H@Ag-NPs are potent anticancer, antibacterial, and antioxidant agents, while H@Au-NPs may be used as a drug vehicle for pharmaceutical applications.

## 1. Introduction

Phyto-mediated synthesis is an emerging cutting-edge nanobiotechnology due to its fast, renewable, and eco-benign synthesis method with low-cost consumption and hazard yields compared to conventional methods [1,2]. Phytosynthesis depends on the potential of plants to uptake, accumulate, utilize, and recycle different mineral species [3,4]. Generally, in an ecosystem, biological systems such as bacteria, diatoms, plants, etc., produce a variety of superstructure inorganic nanomaterials, such as magnetite, amorphous silica, and calcite, to reduce their toxicity via detoxification or remediation processes [5,6,7]. These biological systems contain a vast number of biomolecules, such as proteins, enzymes, pigments, essential oils, fatty acids, and hydrocarbons, and metabolites, such as terpenoids, flavonoids, and others, which work as reductants and capping agents for reducing and stabilizing nanoparticles (NPs) [8,9,10,11]. Due to the necessity to produce large-scale yields of biocompatible NPs with low toxicity to living systems in short time periods, plants are considered the most attractive neutral source for NP synthesis [1]. The use of plants for NP synthesis has many advantages, including availability, safety, high biomass production, and a wide range of primary and secondary metabolites that reduce precursor metals into their NP form in a few minutes or hours [10,12]. Inorganic NPs have acquired significance in various applied sectors, such as in medical, pharmaceutical, industrial, electronic, etc., applications. These NPs are used as therapeutic agents [13], biosensors [14], bioimaging [15], drug carriers [16], and electroactive cells [17]. The wide range of inorganic NP uses arises from their distinct physicochemical and biological characteristics, including their small size to the large surface area, high surface energy, a large fraction of surface atoms, reduced imperfections, spatial confinement, surface plasmon light scattering, surface plasmon resonance (SPR), surface-enhanced Rayleigh scattering, surface-enhanced Raman, and scattering (SERS) properties [18,19]. Many reports have shown the ability of several plant parts, including the leaves, roots, stems [20], seeds [21], flowers [22], and others, to synthesize various controlled shapes and sizes of inorganic NPs that incorporate silver [20], gold [20], platinum [23], palladium [24], and metal oxides such as titanium oxide [25], iron oxide [26], copper oxide [27], etc. These green NPs exhibit potent biological activity against pathogenic bacteria, such as *Streptococcus pneumoniae* causing meningitis [28], *Klebsiella pneumoniae* causing pneumonia and bloodstream infections [29], *Escherichia coli* causing urinary tract infections [30], and *Staphylococcus aureus* causing infective endocarditis and osteoarticular infections [31]. Moreover, biogenic NPs show great antiproliferative activities against cancers, such as breast, cervical, brain, leukemia, colon, etc., at experimental levels [32]. On the other hand, plant-mediated NPs have anti-inflammatory [33], antidiabetic [34], antiparasitic [35], antioxidant [36], and catalytic activities [37]. General NPs enhance apoptotic pathways inside malignant cells via crosstalk between several events, including enhancing ROS formation causing oxidative stress, impacting protein expression, DNA damage, immunological interventions, inhibition of transcription, site-specific cytotoxicity, and others [38]. Similarly, the main mechanism of NP action against bacterial cells is due to their potential to induce oxidative stress and interact with cellular barriers to alter membrane permeability and integrity and impact the functions of cellular components, including DNA, proteins, and enzymes, to cause dysfunction and bacterial cell death [39]. The current study reports for the first time a one-step synthesis method using *Haloxylon salicornicum* aqueous extract to create silver and gold NPs while emphasizing their anticancer, antibacterial, and antioxidant activities.

## 2. Materials and Methods

### 2.1. Materials

Silver nitrate (AgNO_3_), MTT, Resazurin dye, FRAP and DPPH kits, DMSO, and microbial materials such as nutrient agar were purchased from Sigma-Aldrich, St. Louis, MO, USA. Chloroauric acid (HAuCl_4.3_H_2_O) was purchased from Loba Chemie. Cell culture media, trypsin, penicillin/streptomycin, and phosphate buffer saline were purchased from Gibco, Waltham, MA, USA.

### 2.2. Methods

#### 2.2.1. Preparation of Plant Extracts

*H. salicornicum* leaves were collected from the Experimental Research Station, Faculty of Food and Agriculture Sciences, King Saud University, Dirab, 35 km southwest of Riyadh, Saudi Arabia (24°39′ N, 46°44′ E) and washed three times with distilled (dist.) water to remove any undesired matter and dried in an oven at 50 °C for three days. Dried leaves were crushed into a fine powder using a Waring grinder machine (Waring 7011SC Blender, Waring, Stamford, CT, USA) and kept in sterilized containers for further applications. Plant powder (2 g) was mixed with 100 mL of dist. H_2_O and boiled at 80 °C in a water bath for 15 min. After heating, the mixture was allowed to be cool and was filtered using Whatman filter paper No. 1, size 185 mm. The filtrate was centrifuged at 4700 rpm for 10 min to eliminate any plant debris [40].

#### 2.2.2. Gas Chromatography-Mass Spectroscopy

The *H. salicornicum* extract was prepared by mixing 2 g dried powder with 100 mL boiled dist. H_2_O (80 °C) and sonicating for 30 min. The specimen was allowed to macerate for 24 h and centrifuged at 4700 rpm for 10 min. The supernatant was filtered using a syringe filter (0.22 µm), and the filtrate was dried under a vacuum at 50 °C for 48 h. A Trace GC-TSQ mass spectrometer (Thermo Scientific, Austin, TX, USA) with a direct capillary column TG–5MS (30 m × 0.25 mm × 0.25 µm film thickness) was used to identify the phytometabolites in the *H. salicornicum* extract under the same conditions and methods mentioned in our previous published paper [41]. Plant biomolecules were detected by comparison of their mass spectra with those of WILEY 09 and NIST 14 mass spectral databases.

#### 2.2.3. Silver and Gold Nanoparticle Synthesis Using Aqueous Plant Extracts

To fabricate H@Ag-NPs, 1 mL of aqueous plant extract was mixed with 9 mL of 1 mM AgNO_3_ and kept under a light (2000 ± 200 lux) for 24 h at ambient temperature. In this step, the color of the mixture converted from golden yellow to reddish brown after 24 h. H@Au-NPs were synthesized by mixing 1 mL of plant extract with 9 mL of 1 mM of HAuCl_4.3_H_2_O and kept at 80 °C in a water bath for 30 min until the reddish pink color became stable. Afterward, the H@Au-NPs suspension was kept at ambient temperature in the dark for 24 h. After 24 h, H@Ag-NP and H@Au-NP suspensions were centrifuged at 12,000 rpm for 15 min and washed at least three times with dist. H_2_O to remove any undesired plant matrix. Both H@Ag-NP and H@Au-NP pellets were collected and freeze-dried using a lyophilizer (LYOTRAP, LTE Scientific, Greenfield, UK) for 12 h [42,43].

#### 2.2.4. Physicochemical Characterization of Silver and Gold Nanoparticles

##### UV-Vis Spectroscopy

To detect the wavelengths of H@Ag-NPs and H@Au-NPs, an aliquot (2 mL) was drawn from the H@Ag-NP and H@Au-NP suspensions after 24 h and measured using UV-spectroscopy (Model UV-1800, Shimadzu, Kyoto, Japan) at a wavelength range of 200–800 nm with 1 nm resolution.

##### X-ray Diffraction Analysis

The crystalline structures of H@Ag-NPs and H@Au-NPs were detected using an X-ray diffractometer, D8 Advance (Bruker, Bremen, Germany). The dried H@Ag-NP and H@Au-NP powders were coated on XRD grids and estimated over 0° to 80° (2θ) using Cu K α radiation generated at 30 kV and 30 mA with a scan speed 4 deg/min.

##### Fourier-Transform Infrared Spectroscopy

The functional groups of biomolecules surrounding H@Ag-NP and H@Au-NP surfaces that existed in the *H. salicornicum* extract were estimated using FTIR (Shimadzu, Kyoto, Japan). H@Ag-NP and H@Au-NP suspensions were centrifuged at 12,000 rpm for 15 min, washed at least three times, and lyophilized. The powdered H@Ag-NPs, H@Au-NPs, and plant extract were examined within a spectra range of 400 to 4000 cm^−1^ at a resolution of 4 cm^−1^.

##### Transmission Electron Microscope

TEM was used to determine the shape and size of H@Ag-NPs and H@Au-NPs. After washing H@Ag-NPs and H@Au-NPs at least three times with dist. H_2_O, the pellets were washed three times with 70% ethanol, and 200 µL of each NP suspension was kept in 1 mL of ethanol and sonicated for 15 min. Then, 20 µL of each NP suspension was loaded onto carbon-coated copper grids and allowed to dry at room temperature. The samples were examined using TEM (JEM-1400Flash, Jeol, Akishima, Japan) at 120 kV. The frequency distribution of H@Ag-NPs and H@Au-NPs was determined by measuring the diameter of approximately 100 NPs using ImageJ [41].

##### Scanning Electron Microscope, Energy-Dispersive X-ray, and Mapping Analysis

SEM was used to demonstrate the morphological appearance of H@Ag-NPs and H@Au-NPs. An amount of 20 µL of the sonicated NP suspensions was spread onto cleaned glass slides and allowed to dry at room temperature. Dried slides were then coated with platinum using a sputter coater (JEC-3000FC, Jeol, Japan) at 1.8 pa and 10 mA for 80 s, and the glass piece was pasted to a copper stub using carbon paste tape and examined at 15 kV. A JSM-IT500HR EDX detector (STD-PC80, Jeol, Japan) was used to demonstrate the elemental compositions and elemental distribution maps of H@Ag-NPs and H@Au-NPs. NP powders were placed on clean clink paper and transferred to carbon paste strips attached to copper stubs and smoothly knocked to remove any excess powder and coated by platinum [41].

##### Dynamic Light Scattering and Zeta Potential Analysis

The hydrodynamic diameter, particle distribution, and potential charge of H@Ag-NP and H@Au-NP suspensions were detected using Zetasizer equipment (Malvern, UK). In brief, H@Ag-NP and H@Au-NP suspensions were diluted ten-fold using dist. H_2_O and then sonicated for 15 min and transferred into Zetasizer tubes at 25 °C for examination.

#### 2.2.5. Antimicrobial Activity of Silver Nanoparticles

##### Agar Well Diffusion Method

The agar well diffusion method was performed to detect the biocidal activity of *H. salicornicum*, H@Ag-NPs, H@Au-NPs, and ciprofloxacin (as positive control) against four pathogenic bacteria, including two Gram-positive (*Staphylococcus aureus* ATCC 29213 and *Bacillus cereus* ATCC 9634), and two Gram-negative bacteria (*Escherichia coli* ATCC 25922 and *Klebsiella pneumoniae* ATCC13883). The tested bacteria were cultured in nutrient broth for up to 18 h at 37 °C and maintained through continuous subculturing in broth and on solid media. Bacterial isolate (4 mL of 2.5–3.6 × 10^6^ CFU/mL) was mixed with 50 mL nutrient agar medium, poured into sterilized Petri dishes, and dried at 37 °C. Four 8 mm wells were created in the agar plates using a cork borer. After that, 100 µL of ciprofloxacin (5 µg/mL as a positive control), 25 mg/mL *H. salicornicum* extract, or 1 mg/mL of H@Ag-NPs and H@Au-NPs was added to each well. An amount of 100 µL dist. H_2_O served as a negative control. The plates were incubated for 24 h at 37 °C, and the inhibition zone of each treatment was measured using a transparent ruler [44].

##### Minimum Inhibition and Maximum Bactericidal Concentrations

A resazurin dye assay was performed to determine the minimum inhibition concentration at which 99% of cell growth was inhibited by the treatment and the minimum bactericidal concentration at which 100% of cell growth was inhibited. A total of 100 µL of two-fold serial dilutions of *H. salicornicum*, H@Ag-NPs, and H@Au-NPs (500, 250, 125, 62.5, 31.25, 15.62, 7.8, 3.9, 1.95, and 0.98 µg/mL) were added in triplicate across 96-well plates (from column 1 to 10). Next, 100 µL of the bacterial suspension (2.5–3.6 × 10^6^ CFU/mL) was mixed into each well; column 11 represented the positive control (bacterial suspension without treatment), and column 12 represented the negative control (media only). The plates were kept in the incubator for 24 h at 37 °C. After incubation, 30 µL/well of resazurin dye solution (0.015 g resazurin in 100 mL dist. H_2_O) was added, and plates were incubated at 37 °C for 4 h. Absorbance was then measured using a plate reader at 570 nm. Columns with a resazurin blue color (unchanged) were considered to be above the MIC value. The MBC values were estimated by plating the contents of the wells with concentrations higher than the MIC value on nutrient agar plates [45].

#### 2.2.6. Anticancer Activity of Silver Nanoparticles

##### Cell Culture

The selected cell lines (colon cancer cell lines; Sw480, Sw620, HCT-116, Caco-2, and normal cell lines; human fibroblast (HFs) and kidney cells of an African green monkey (Vero)) were cultured in complete RPMI and DMEM media containing 10% FBS and 50 U/mL penicillin and streptomycin antibiotics. The cells were kept in a 5% CO_2_ incubator at 37 °C. At 80% confluency, the cells were trypsinized using trypsin-EDTA and counted, and 5 × 10^4^ cells/well seeded into 96-well plates and kept in a 5% CO_2_ incubator for 24 h at 37 °C [41].

##### MTT Assay

An MTT assay was used to detect the cytotoxicity of H@Ag-NPs, H@Au-NPs, and 5-FU (positive control) against the selected cells. Firstly, 500 µg of H@Ag-NPs and H@Au-NPs were weighed and dissolved in 1 mL of DMEM media and sonicated for 15 min until all particles became well dispersed in the media. After that, the suspension was filtrated using a microfilter with a pore size of 0.45 µm. Several concentrations of filtrated 5-FU, H@Ag-NPs, and H@Au-NPs (500, 250, 125, 62.5, 31.25, 15.62, 7.8, and 3.9 µg/mL) were prepared, and 100 µL of each concentration was added to cells in 96-well plates and incubated at 37 °C for 24 h. The media of treated plates were discarded, and 100 µL/well of fresh media was added to each well. Then 10 µL/well of MTT solution (5 mg/mL) was mixed with media and kept in the dark at 37 °C for 4 h. After incubation, 100 µL of absolute DMSO was added to each well to dissolve the formazan crystals and kept on a shaker plate (120 rpm) for 15 min. The absorbance was detected using an ELISA plate reader (Bio-Rad, Hercules, CA, USA) at 570 nm [46]. The cell viability (%) was estimated according to the following equation

Abs_(treated)_ − Abs_(blank)_/(Abs_(control)_ − Abs_(blank)_) × 100

The IC_50_ (half-maximal growth inhibitory concentration) was calculated using a sigmoidal curve.

#### 2.2.7. Antioxidant Activity of Nanoparticles

##### DPPH Assay

The scavenging activity of plant extracts, H@Ag-NPs, and H@Au-NPs was assessed using a DPPH assay. In brief, 1 mg of each ascorbic acid, plant extract, H@Ag-NPs, and H@Au-NPs were dissolved in 1 mL absolute methanol and vortexed (1 min for ascorbic) or sonicated for 15 min (for NPs). Ascorbic acid, H@Ag-NPs, and H@Au-NPs were serially diluted two-fold in 96-well plates (1000, 500, 250, 125, and 62.5 µg/mL). A blank well was set as the negative control. An amount of 100 µL/well of DPPH (0.004 g/mL) was added, and the plates were incubated for 30 min in the dark. After that, the plates were shaken using a plate shaker for 1 min, and the absorbance was recorded at 517 nm using an ELISA plate reader (Bio-Rad, USA) [41].

##### FRAP Assay

The antioxidant activity of 2 mg/mL of *H. salicornicum*, H@Ag-NPs, and H@Au-NPs was examined using a FRAP assay. The 2,4,6-Tri(2-pyridyl)-s-triazine (TPTZ) reagent was freshly prepared (300 mM acetate buffer, pH = 3.6), and 10 mM TPTZ was suspended in 40 mM HCl and 20 mM FeCl_3_ at a ratio of 10:1:1 (*v:v:v*), respectively. In a 96-well plate, 10 µL of each treatment was mixed with 190 µL of TPTZ. The plates were incubated in the dark at ambient temperature for 30 min. Afterward, the plates were read using a plate reader at 593 nm [20,47].

#### 2.2.8. Statistical Analysis

Data were collected from three independent triplicates and are presented as the mean ± SEM. Statistical significance was determined using GraphPrism software version 9 via one-way ANOVA analysis. ImageJ software (National Institutes of Health, Bethesda, MD, USA) was used to measure NP particle diameter, and Origin 8 (OriginLab Corporation, Northampton, MA, USA) was used to draw all physicochemical analysis data.

## 3. Results

### 3.1. GC-MS Analysis of Haloxylon Salicornicum

**Figure 1:**
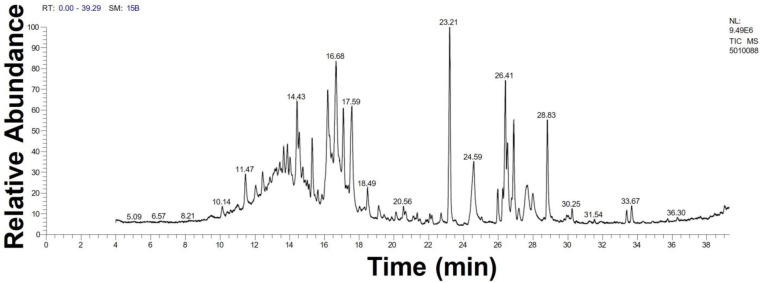
GC-MS chromatograph illustrating the chemical composition of the *Haloxylon salicornicum* aqueous extract.

The GC-MS analysis demonstrated that 51 peaks for *H. salicornicum* correlated to 36 biomolecules (Figure 1 and Table 1). These 36 volatile phytocomponents included terpenes and their derivatives: alcohol, aldehyde, and ketone of terpenoids, fatty acids and their ester, aliphatic and aromatic hydrocarbons, and carboxylic acids. These derivatives may act as reducing and capping agents during the synthesis process for H@Ag-NPs and H@Au-NPs [48]. Monoterpenoids and sesquiterpenoids are potent contributors in Ag-NP synthesis and may act as surface activators that participate in the reduction or/and stabilization process of NPs [49,50]. Moreover, the existence of functional groups, such as C=C, C-C, C-O, –C=O, CH_2_, CH_3_, –OH, C-H, CH_3_-C-CH_3_, –C–O–C and –C–N groups, in the terpenoid structure determine their role as reductants and stabilizers [51,52,53,54]. Many *H. salicornicum* biomolecules are important plant metabolites, such as 1-methyl-2-(1-methylethenyl)-4-(1-methylethylidene)-1-vinylcyclohexane), (1R-trans)-, cyclohexane, 1-ethenyl-1-methyl-2,4-bis(1-methylethenyl)-, (1R,2R,4S)-rel-, beta-longipinene; anti-inflammatory agents, such as aromandendrene; and antioxidant agents such as alpha-copaene and (-)-globulol, etc. [55,56]. Ullah et al. reported that *H. salicornicum* extracts contain the biomolecules 5-butoxy-2-pentene (55.510%), 2,3-dihydro-3,5-dihydroxy-6-methyl-4h-pyran-4-one (14.640%), D-allose (10.080%) and oxalic acid [57]. Our findings showed that *H. salicornicum* extracts contain a variety of plant metabolites that may have therapeutic activity against different diseases.

### 3.2. Ag-NP and Au-NP Synthesis

**Figure 2:**
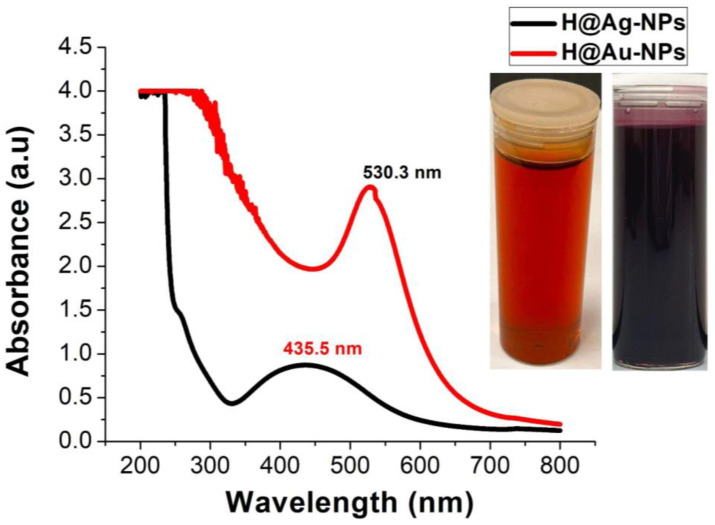
UV-Vis spectroscopy graphs of H@Ag-NPs and H@Au-NPs synthesized by *Haloxylon salicornicum*.

During H@Ag-NP synthesis, the color of the solution transformed from pale yellow to dark yellow after 1 h of incubation in the light, then to faint reddish brown after 2 h. Finally, the solution turned a dark reddish-brown color that was stable after 24 h (Figure 2). The color of the H@Au-NPs was reddish pink after 1 h of synthesis and was stable during the incubation time. The wavelengths of H@Ag-NPs and H@Au-NPs were 435.5 nm [58] and 530.3 nm [59], respectively, indicating the successful synthesis of both NPs. The wavelength of Ag-NPs ranged between 400 and 450 nm [60], while Au-NPs ranged from 500 to 550 nm [61]. The maximum SPR for NPs depends on the electron charge density on the NP surface, particle size, shape, metal type, composition, etc. [62]. Smaller size NPs showed a decrease in their SPR values due to the phase changes resulting from the increased rate of electron-surface collisions compared to larger particles. Increments in NP size shift their wavelength and surge their intensity. The SPR of both H@AgNPs and H@Au-NPs indicate that these NPs have a median NP size. Chelly et al. synthesized Ag- and Au-NPs using a methanolic extract of *Rumex roseus*, which have SPR at 429 and 549 nm, respectively [63]. Lomelí-Rosales et al. synthesized Ag- and Au-NPs using aqueous extracts from the root, stem, and leaf of *Capsicum chinense* [20]. It was found that the SPR of Au-NPs synthesized using root, stem, and leaf was 553 nm, 548 nm, and 523 nm, respectively, whereas the SPR of Ag-NPs synthesized using the leaf was 430 nm. The scholar established the stability assay and reported that the stability of Ag-NPs was higher than that of Au-NPs.

#### 3.2.1. XRD Analysis

**Figure 3:**
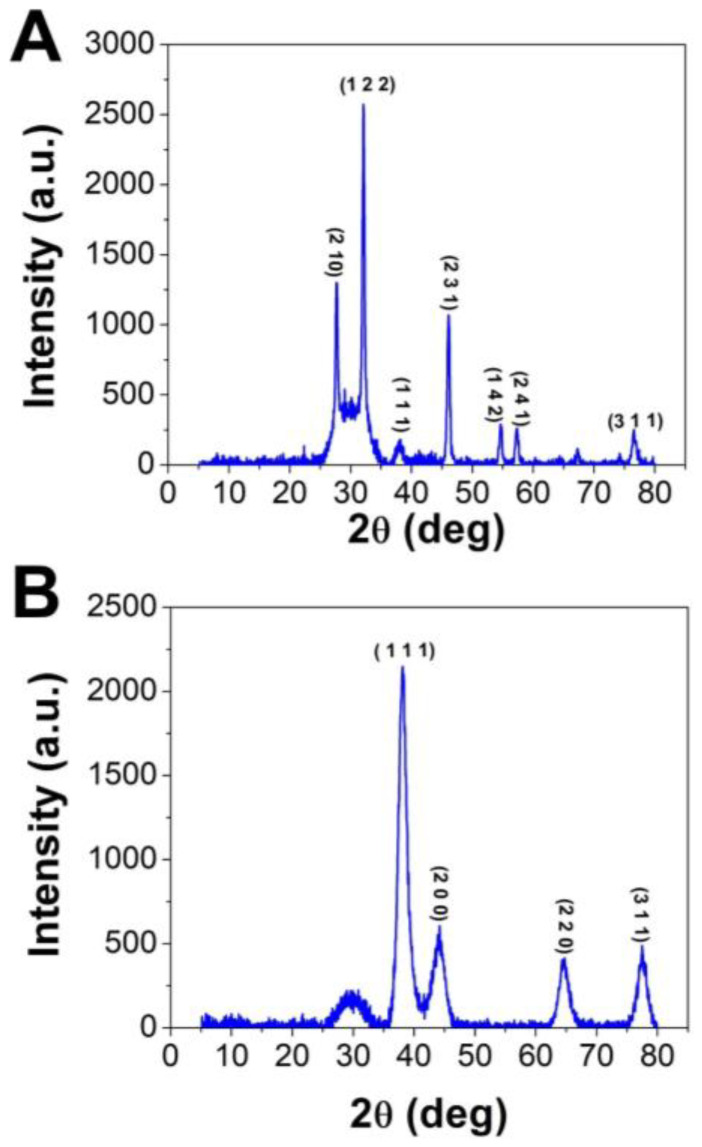
XRD graphs of H@Ag-NPs (**A**) and H@Au-NPs (**B**) synthesized using *Haloxylon salicornicum*.

XRD analysis was performed to detect the crystalline nature and crystal size of both H@Ag- and H@Au-NPs. The diffractogram has been compared with the standard powder diffraction card of JCPDS silver and gold files no. 04-0783 and 04-0784, respectively. The XRD graphs of H@Ag-NPs exhibited six diffraction peaks at 2θ of 27.86°, 32.18°, 38.1°, 46.1°, 54.6°, 57.4°, and 76.7°, corresponding to 2 1 0, 1 2 2, 1 1 1, 2 3 1, 1 4 2, 2 4 1, and 311. H@Au-NPs exhibited four diffraction peaks at 2θ of 38.4°, 44.2°, 64.5°, and 77.4°, corresponding to 1 1 1, 2 0 0, 2 2 0, and 3 1 0. These data indicate the crystallinity of both H@Ag-NPs and H@Au-NPs (Figure 3). The Scherrer equation, D = (kλ)/(β cos θ), was utilized to detect the crystallite size (D) of the most intense peak at 2ϴ of 32.0 and 38.2 for H@Ag-NPs and H@Au-NPs, respectively. H@Ag-NPs and H@Au-NPs crystallite sizes (D) were found to be 16.0 and 5.8 nm, respectively. The crystal size values of both NPs were in accordance with the nanosize of H@Ag-NPs and H@Au-NPs measured by ImageJ software using TEM micrographs. Arshad et al. synthesized Ag-NPs using *Salvadora persica* root extract and reported their 2θ at 27.86°, 32.34°, 38.2°, 46.2°, 54.74°, 57.3°, and 76.72°, which correlate to the lattice planes of 210, 122, 111, 231, 142, 241, and 311 [64]. Ssekatawa et al. synthesized Ag-NPs using *Camellia sinensis* extract and found that the XRD graphs had nine peaks at 2θ of 27.9°, 32.2°, 38.2°, 44.4°, 46.3°, 54.8°, 57.6°, 64.5°, and 77.4° [65]. The authors reported that 38.2°, 44.4°, 64.5°, and 77.4° corresponded to the crystal planes of Ag-NPs at 111, 200, 220, and 311, while the other peaks corresponded to phytochemicals on the NP surface. Au-NPs synthesized using methanolic extracts from *Moringa oleifera* leaves had XRD graphs at 2θ of 38.3, 44.4, 64.5, and 77.5 that corresponded to 111, 200, 220, and 311 of the face-centered cubic lattice [43].

#### 3.2.2. FTIR

**Figure 4:**
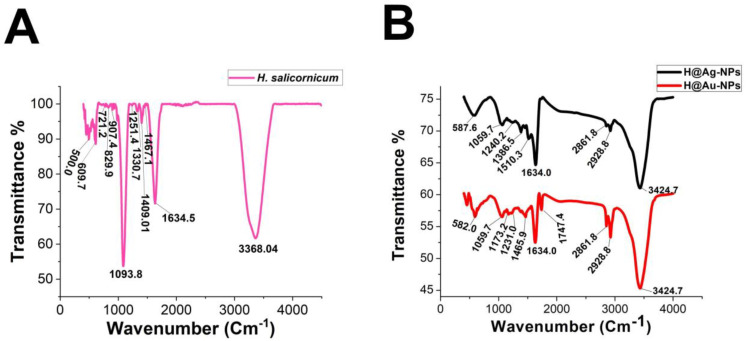
FTIR spectra of *Haloxylon salicornicum* extract (**A**) and H@Ag-NPs and H@Au-NPs (**B**).

FTIR demonstrated that the functional groups of biomolecules exist in the plant extract, H@Ag-NPs, and H@Au-NPs (Figure 4 and Table 2). The IR peaks of *H. salicornicum* extract showed 12 peaks at 3368.04, 1634.5, 1467.1, 1409.01, 1330.7, 1251.4, 1093.8, 907.4, 829.9, 721.2, 609.7, and 500.0 cm^−1^. IR peaks of 3368.04 and 1634.5 cm^−1^ corresponded to strong, broad O-H stretching of alcohol and medium N-H bending of amines, respectively, while peaks at 1467.1 and 1409.01 cm^−1^ represented medium C-H bending of alkane and medium O-H bending of alcohol or carboxylic acid, respectively. FTIR peaks at 1330.7, 1251.4, and 1093.8 were related to strong C-N stretching of aromatic amine, strong C-O stretching of aromatic ester or alkyl aryl ether, and strong C-O stretching of a secondary alcohol. Peaks at 907.4, 829.9, 721.2, 609.7, and 500.0 cm^−1^ corresponded to strong C=C bending alkene, strong C-Cl stretching of halocompound, strong C=C bending of alkene, strong C-Cl and C-I stretching of halocompound, respectively. The IRs of H@Ag-NPs were located at nine peaks of 3424.7 [66], 2928.8 [67], 2861.8 [68], 1634.0 [69], 1510.3, 1386.5, 1240.2, 1059.7 [70], and 587.6 cm^−1^, corresponding to strong, broad O-H stretching of alcohol; medium C-H stretching of alkane; medium C-H stretching of alkane; medium C=C stretching of alkene; strong N-O stretching of nitrocompound; medium C-H stretching of aldehyde; medium C-N stretching of amine; strong C-O stretching of primary alcohol; and strong C-Cl stretching of halocompound. FTIR peaks of H@Au-NPs occurred at 3424.7, 2928.8, 2861.8, 1747.4 [68], 1634.0, 1465.9 [68], 1231.0 [70], 1173.2 [71], 1059.7 [70], and 582.0 cm^−1^, corresponding to strong, broad O-H stretching of alcohol; medium C-H stretching of alkane; medium C-H stretching of alkane; strong C=O stretching of esters or δ-lactone; strong C=C stretching of alkene; medium C-H bending of alkane; medium C-N stretching of amine; strong C-O stretching of tertiary alcohol or ester; strong C-O stretching of primary alcohol; and strong C-Cl stretching of halocompound. By analyzing the IR spectra of both plant extract and NPs, it was found that the main functional groups on the NPs’ surface were O-H or C-O of alcohol, N-O of nitrocompound, C-N of amine and C-H of hydrocarbons (essential oils/terpenes), suggesting that these biomolecules might mitigate the synthesis process of NPs as reducing and capping agents [72]. Interestingly, the IR of H@Ag-NPs and H@Au-NPs have the same spectra at 3424.7, 2928.8, 2861.8, 1634.0, and 1059.7 cm^−1^, suggesting that these biomolecules might be the main and essential plant metabolities (defenders) responsible for fabricating the bulk materials into nanoscale matter. Moreover, the existence of other spectra rather than the main components on both NP surfaces varied depending on the type, composition, and structure of the metals. For instance, the surface of H@Ag-NPs was found to have three other spectra, including 1510.3, 1386.5, and 1240.2 cm^−1^, whereas H@Au-NPs had four spectra, 1747.4, 1465.9, 1231.0, and 1173.2 cm^−1^. This variation in the functional groups on the NP surface may be a response to the physicochemical characteristics of the metals. The IR spectra was in correspondence with data from the GC-MS analysis, in which the volatile phytocomponents of *H. salicornicum* were terpenes and its derivative alcohol, aldehyde, ketone of terpenoids, fatty acids and its ester, aliphatic and aromatic hydrocarbons, and carboxylic acids with the main functional groups of C=O, C-O, C-H, O-H, C=N, C-Cl, etc. 

#### 3.2.3. Transmission Electron Microscope

**Figure 5:**
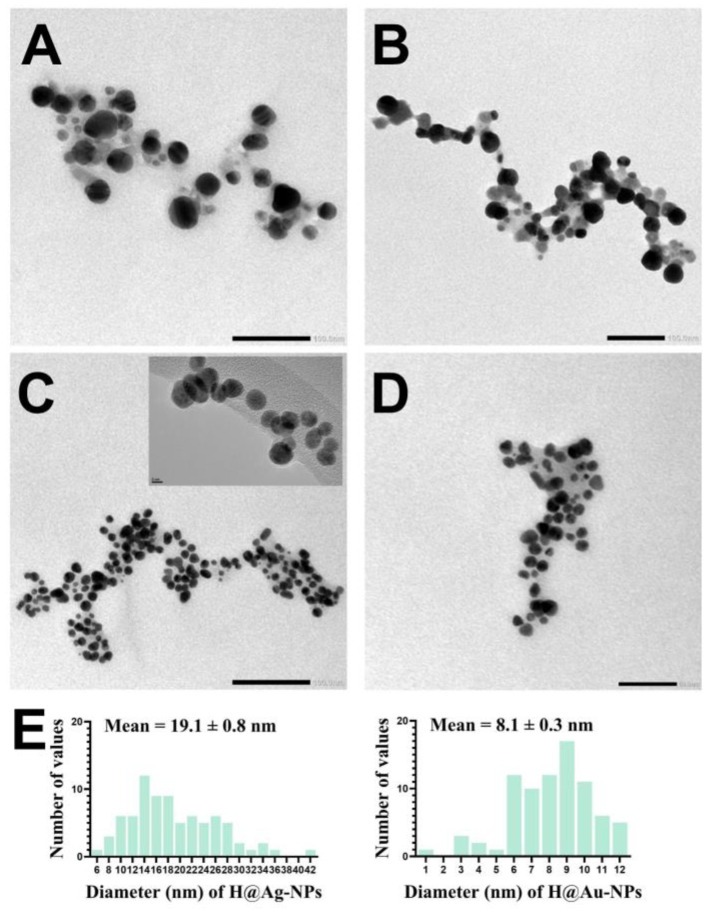
TEM micrographs of H@Ag-NPs (**A**,**B**) and H@Au-NPs (**C**,**D**) and their frequency distribution (**E**). Scale bar of (**A**,**B**) at 100 nm and (**D**) at 50 nm.

TEM micrographs showed that H@Ag-NPs have a uniform spherical shape coated with plant matrix (organic compounds), whereas H@Au-NPs have a spherical shape with a few oval and triangular shapes (Figure 5A–D). The frequency distribution of both H@Ag-NPs and H@Au-NPs demonstrated that they have an average nanodiameter of 19.1 ± 0.8 nm and 8.1 ± 0.3 nm and nanosize range of 6 to 42 and 1 to 12 nm, respectively (Figure 5E). Singh et al. synthesized Ag-NPs and Au-NPs using *Ligustrum vulgare* berry extract and reported that Ag-NPs and Au-NPs have various shapes with nanosize ranges of 50–200 and 20–70 nm, respectively [73].

#### 3.2.4. Scanning Electron Microscope and EDX

**Figure 6:**
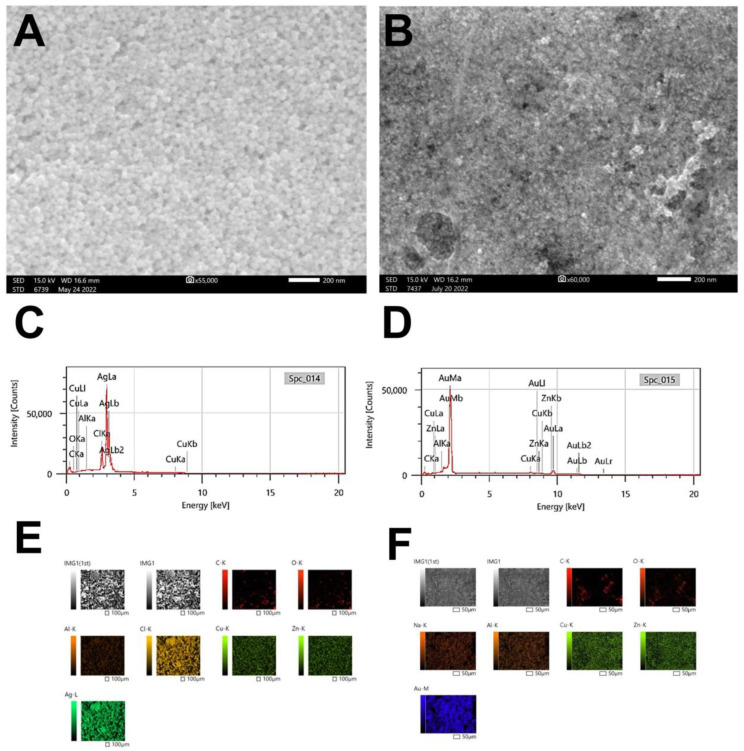
Scanning electron microscopy micrographs (**A**,**B**) and energy diffraction X-ray (**C**,**D**) and map analyses (**E**,**F**) of H@Ag-NPs and H@Au-NPs, respectively. Scale bar of (**A**,**B**) at 200 nm.

SEM micrographs showed that both H@AgNPs and H@Au-NPs have spherical shapes (Figure 6A,B). EDX and mapping analyses revealed that the dominant chemical composition of H@AgNPs was silver (Ag; 88.61%) at 3 keV [41]. Other elements were detected, including chloride (7%), carbon (2.5%), copper (1%), oxygen (0.58%), and aluminum (0.14%).

The most distributed element in H@Au-NP samples was Au at 2.3 and 9.7 keV (85%), followed by copper (5.5%), carbon (3.9%), zinc (3.7%) and aluminum (0.21%) (Figure 6C–F and Table 3) [74]. Additionally, map analysis exhibited other trace elements, including oxygen and Na, in the H@Au-NPs sample. It was found that C, Cl, O, etc., are impurities derived from plant materials and coated NPs; these elements are important for plant growth and metabolism.

#### 3.2.5. Zeta Potential and DLS Analysis

**Figure 7:**
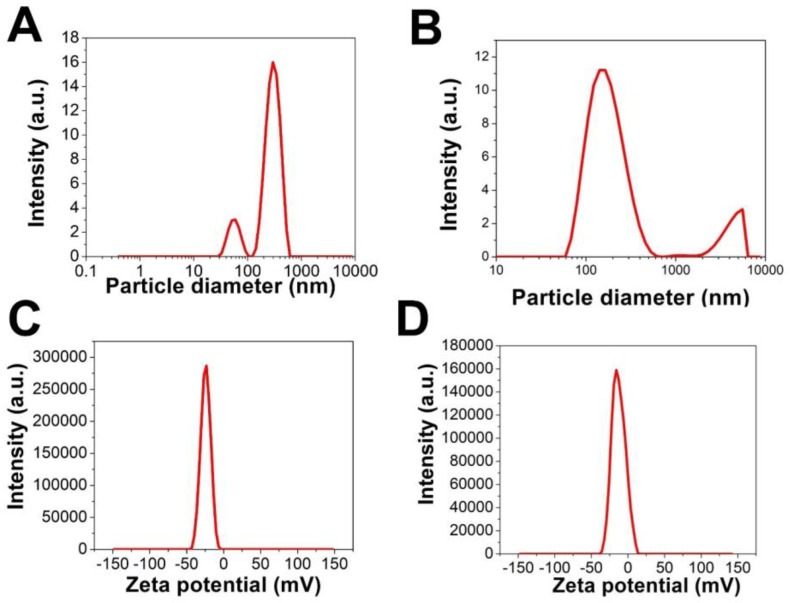
Dynamic light scattering (**A**,**B**) of H@Au-NPs and H@Ag-NPs, respectively, and zeta potential (**C**,**D**) graphs of H@Au-NPs and H@Ag-NPs, respectively.

The zeta potential of H@Ag-NPs and H@Au-NPs was −24.0 and −24.4 mV, respectively, whereas the H@Ag-NP hydrodynamic diameter (HD) was 184.7 nm with a polydispersity index (PDI) of 0.411. H@Au-NPs had two HDs at 295.4 and 56.4 nm with a PDI of 0.47 (Figure 7A–D). The great negativity of both H@Ag-NPs and H@Au-NPs indicated that these NPs demonstrate colloidal stability; however, the high HD values for both H@Ag-NPs and H@Au-NPs may be due to saturated biomolecules and water molecules of the aqueous system coating the NPs. Ag- and Au-NPs fabricated using *Ligustrum vulgare* had HDs of 542.6 nm with a PDI of 0.479 and 292.3 nm with a PDI of 0.3, with zeta potentials of −18.7 and −20.8 mV, respectively [73].

### 3.3. Anticancer Activity of Ag-NPs

**Figure 8:**
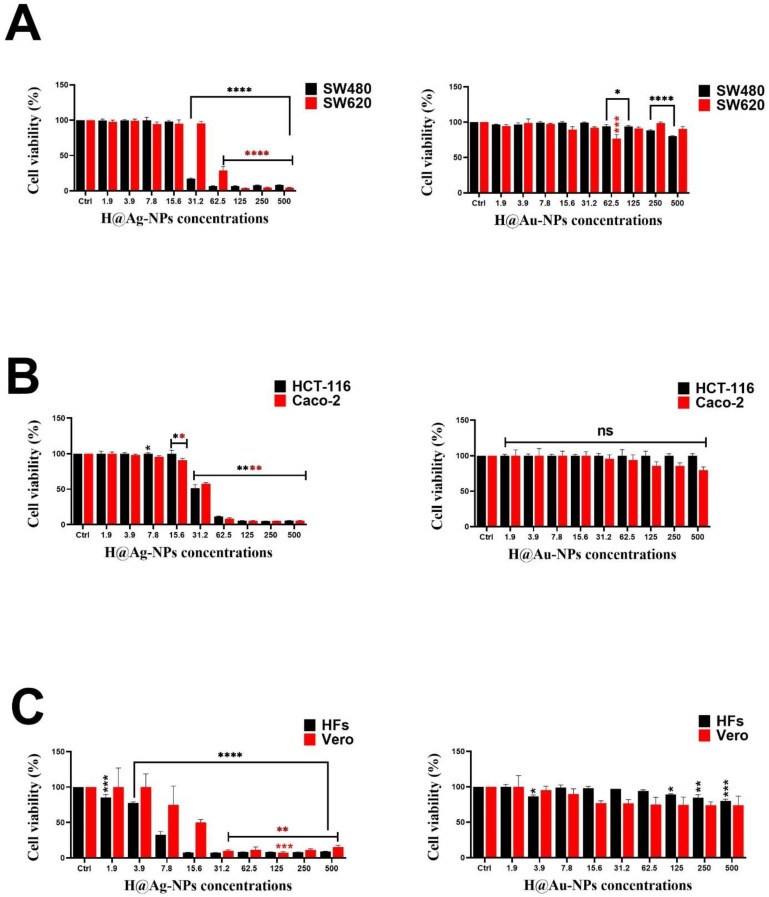
Antiproliferative activity of H@Ag-NPs and H@Au-NPs against (**A**) Sw480 and Sw620, (**B**) HCT-116 and Caco-2, and (**C**) HFs and Vero. Data are shown as the mean ± SEM. *p*-values were calculated using untreated cells as the control. **** *p* < 0.0001, *** *p* < 0.0002, ** *p* < 0.001 and * *p* < 0.01. ns refers to non-significance.

**Figure 9:**
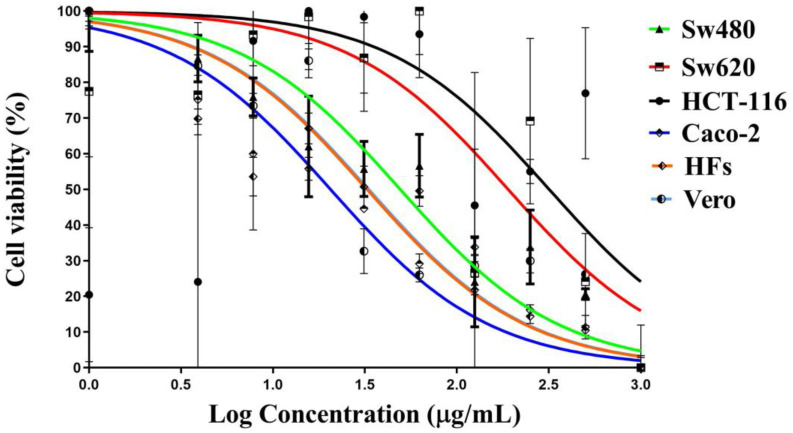
Cell viability % of 5-FU against colon cancer cells Sw480 and Sw620, HCT-116 and Caco-2, and normal cells HFs and Vero cells. Data are shown as the mean ± SEM.

The antiproliferative activity of H@Ag-NPs in the four colon cancer cell lines (Sw480, Sw620, HCT-116, and Caco-2) was higher than that of H@Au-NPs and 5-FU. H@Ag-NPs caused 50% inhibition in the growth of Sw480, HCT-116, Caco-2, and Sw620 cells at concentrations of 20.4, 28.5, 29.8, and 50.23 µg/mL in the respective cell lines. The IC_50_ of H@Au-NPs against all tested malignant cell lines was above the tested concentration of 500 µg/mL. Although H@Ag-NPs were cytotoxic to the malignant cells, they also had a cytotoxic effect on nonmalignant cells with an IC_50_ of 5.0 and 9.0 µg/mL against HFs and Vero, respectively (Figure 8A–F). The IC_50_ of 5-FU against Sw480, Sw620, HCT-116, Caco-2, HFs, and Vero was 49.2, 190.0, 317.5, 20.57, 32.4, and 33.12, respectively (Figure 9). Intriguingly, Sw480 was the most sensitive to H@Ag-NPs, whereas Sw620 was the least permeable to H@Ag-NPs. Both HCT-116 and Caco-2 had similar responses to the same dose of H@Ag-NPs. These data indicate that the antiproliferative activity of H@Ag-NPs was cell type-dependent, which may be due to the differences in the cellular metabolic state that influences the cellular charge and interactions with charged NPs. The greater toxicity of H@Ag-NPs could be due to the higher reactivity of silver ions, which enables them to interact with cellular molecules such as proteins, enzymes, and antioxidants to induce intracellular oxidative stress resulting in enhanced apoptosis. H@Au-NPs showed low toxicity against all malignant and nonmalignant cells at 500 µg/mL. Only 20% of Sw480 cells were inhibited at 500 µg/mL, while 23% of Sw620 cells died at 62.5 µg/mL. H@Au-NPs suppressed the growth of Caco-2 cells by 20% at 500 µg/mL and showed no effect on HCT-116 cells (100% viability at 500 µg/mL). H@Au-NPs inhibited 20% and 16% of HF and Vero cell growth at 500 µg/mL, respectively. The biocompatibility of H@Au-NPs against malignant or nonmalignant cells may be due to their biological inertness or/and the ability of the various cell types to regulate cellular uptake of the H@Au-NPs. Moreover, the low percentage of cell death caused by H@Au-NPs may be due to the plant-derived corona coating the H@Au-NP surface and not due to the Au-NP itself. Conversely, the low antiproliferative activity percentage may be due to the low concentration of corona biomolecules. The concentration may not have been high enough to enhance cellular toxicity. The low antiproliferative activity percentage also may have been due to the absence of active metabolites with significant anticancer activity on the NP surface. Au-NPs have a high surface free energy that enables them to absorb/desorb other molecules, resulting in protein corona formation. These coronas are identified by cells and play a significant role in enhancing the biological activity of NPs by interacting with other molecules in the biological fluids and extracellular matrix. These interactions consequently influence the cellular uptake, biodistribution, signaling, circulation lifetime, and toxicity of the NP [75,76]. Ag- and Au-NPs synthesized using *Commelina nudiflora* aqueous leaf extracts showed antiproliferative activity against HCT-116, with an IC_50_ of 200 and 100 µg/mL [77], whereas Ag-NPs synthesized using *Cornus officinalis* extract showed poor toxicity against Sw620 cell lines. Baran et al. reported that the IC_50_ value for the Ag-NPs synthesized using *Cicer arietinum* L. occurred at a concentration of 200 mg/mL in Caco-2 cell lines [78]. 

### 3.4. Antimicrobial Activity of Ag-NPs

**Figure 10:**
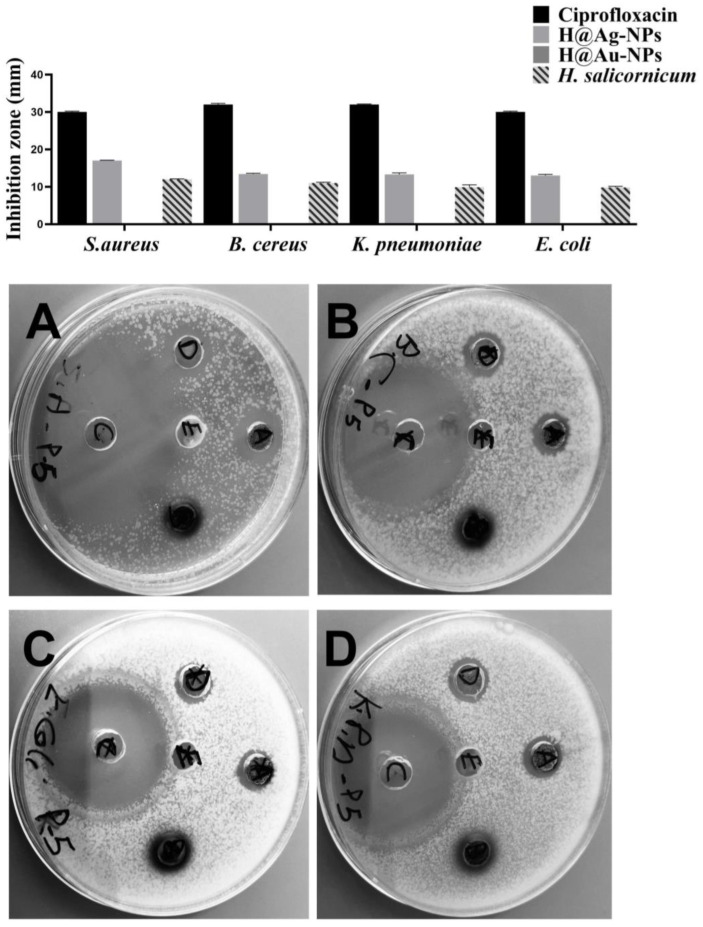
Biocidal inhibitory influence of H@Ag-NPs and H@Au-NPs and *H. salicornicum* aqueous extract against (**A**) *Staphylococcus aureus*, (**B**) *Bacillus cereus*, (**C**) *Escherichia coli*, and (**D**) *Klebsiella pneumoniae*. Written letters on plates refer to (**A**) H@Ag-NPs, (**B**) H@Au-NPs, (**C**) ciprofloxacin, and (**D**) *H. salicornicum* aqueous extract.

The biocidal activity of H@Ag-NPs, H@Au-NPs, *H. salicornicum* extract, and ciprofloxacin was tested against *S. aureus*, *B. cereus*, *E. coli*, and *K. pneumoniae* using agar well diffusion and microdilution methods. The results of MIC and MBC revealed that H@Ag-NPs had the greatest antibacterial and inhibitory activity at a low concentration against the tested bacteria when compared to the other treatments (Table 4). The highest MIC and MBC of H@Ag-NP values were reported against *E. coli* (7.8 and 15.6 µg/mL), followed by *K. pneumoniae* and *B. cereus* (3.9 and 7.8 µg/mL), then *S. aureus* (1.9 and 3.9 µg/mL). These data demonstrate that *S. aureus* was the most permeable to H@Ag-NPs compared to other tested microbes. An amount of 500 µg/mL of H@Au-NPs and *H. salicornicum* extract was not adequate for inhibiting bacterial growth. The agar well diffusion data showed that the biocidal activity of the tested treatments could be represented as follows: ciprofloxacin > H@Ag-NPs > *H. salicornicum* extract > H@Au-NPs (Figure 10). An amount of 1000 µg/mL H@Ag-NPs had great inhibitory activity against Gram-positive bacteria compared to Gram-negative bacteria, and *S. aureus* (17.0 ± 0.1 mm) was the most sensitive to H@Ag-NPs, followed by *B. cereus* (13.4 ± 0.2 mm), *K. pneumoniae* (13.3 ± 0.4 mm), and *E. coli* (13.0 ± 0.0 mm). The activity of 1000 µg/mL H@Ag-NPs against the tested bacteria was half that of ciprofloxacin against the same bacteria (Table 5). The greater activity of H@Ag-NPs against Gram-positive vs. Gram-negative bacteria may be attributed to the nature of the NPs’ charges, in which the negative charge of H@Ag-NPs allows the electrostatic attraction to positively charged bacterial cell walls more than negatively charged bacterial cell walls [79]. Consequently, more adsorbed NPs on the bacterial membrane cause local stress on the bacterial cell wall, resulting in membrane disruption and allowing entrance to more NPs that cause cellular dysfunction via interactions with DNA, proteins, and enzymes and subsequent cell death [80]. On the other hand, 1000 µg/mL H@Au-NPs showed no inhibitory activity (zero IZ) against both Gram-positive and Gram-negative bacteria. These results may be attributed to i) the Au-NPs being biologically inert and having low or no biocidal activity against microbes, or ii) the smaller size of H@Au-NPs (2 to 12 nm), which may enable bacterial cells to efflux NPs or decrease NP permeability and cellular entry [81]. An amount of 20 mg/mL of *H. salicornicum* aqueous extract had lower biocidal activity compared to H@Ag-NPs. The antibacterial activity of *H. salicornicum* was greater against Gram-positive bacteria than Gram-negative bacteria. The greatest activity of *H. salicornicum* was against *S. aureus* (12.0 ± 0.10 mm), while the lowest activity was against both *E. coli* (9.8 ± 0.3 mm) and *K. pneumoniae* (9.9 ± 0.6 mm) (Table 5). Gram-negative bacteria have rigid, thick, and selectively permeable cell walls, enabling these bacteria to mitigate the access of many therapeutic molecules into the bacterial cells [82,83]. Thus, Gram-negative bacteria may be less permeable to plant extracts when compared to Gram-positive bacteria. Moreover, the pharmacokinetics and pharmacodynamics of the plant biomolecules inside the bacterial cells regulate their therapeutic activity.

The biocidal activity of H@Ag-NPs was greater than that caused by plant extracts against the same microbes. This may be attributed to the drug delivery capacity of Ag-NPs to deliver plant biomolecules to the target cells to increase the probability of inducing cell death pathways. Moreover, the therapeutic potential of Ag ions itself may be a significant cause of bacterial growth inhibition. Ag- and Au-NPs synthesized using *Capsicum chinense* have been tested against *S. aureus*, *Enterococcus faecalis*, *E. coli,* and *Serratia marcescens* [20]. In previous studies, Au-NPs showed no antibacterial activity, whereas 4.0 mM of Ag-NPs inhibited *S. aureus*, *E. faecalis*, *E. coli*, and *S. marcescens* growth with an IZ diameter of 10.22 ± 0.46, 5.38 ± 0.26, 8.64 ± 0.12, and 9.96 ± 0.43 mm, respectively. The antibacterial influence of purified Ag-NPs synthesized using *Morinda citrifolia* L. was also tested against *E. coli*, *P. auerginosa*, *K. pneumoniae*, *E. aerogens*, *B. cereus*, and *Enterococcus* sp. and was found to inhibit bacterial growth of *E. coli*, *P. auerginosa*, *K. pneumoniae*, *E. aerogens*, *B. cereus*, and *Enterococcus* sp. with an IZ of 7, 8, 0, 7, 0, and 7 mm. Plant extract showed 0 IZ against all tested bacteria [84].

### 3.5. Antioxidant Activity of Ag-NPs

**Figure 11:**
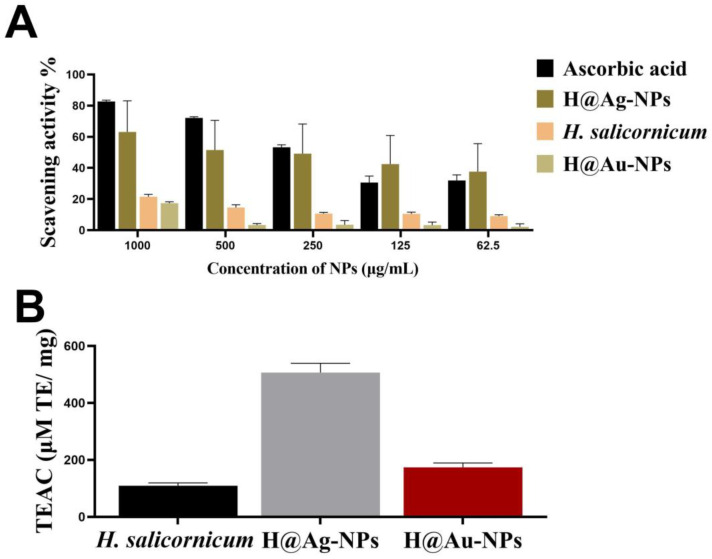
The scavenging activity of H@Ag-NPs and H@Ag-NPs and *H. salicornicum* aqueous extract using FRAP analysis (**A**) and DPPH (**B**) assays.

The scavenging activity of *H. salicornicum* extract, H@Ag-NPs, and H@Au-NPs was examined using FRAP and DPPH assays. The FRAP data revealed that among the tested treatments, H@Ag-NPs have the highest antioxidant activity with TEAC of 506.6 ± 32.7 μM TE/mg, followed by H@Au-NPs and plant extract with TEAC of 173.75 ± 15.3 and 109.4 ± 9.7 μM TE/mg, respectively (Figure 11A). Similarly, the DPPH assay revealed that the scavenging activity of *H. salicornicum* extract, H@Ag-NPs, and H@Au-NPs occurred in a dose-dependent manner. An amount of 1 mg/mL H@Ag-NPs had the greatest scavenging activity (63.1%) compared to H@Au-NPs (17.4%) and plant extract (21.5%) (Figure 11B). The greater antioxidant activity of H@Ag-NPs compared to other treatments may be due to the higher reactivity and surface chemistry of silver ions that enable the binding of more bioactive antioxidant molecules on the NPs’ surface. Lomelí-Rosales et al. determined the antioxidant activity of plant extracts and their biosynthesized Ag- and Au-NPs using ABTS, FRAP, and DPPH assays [20]. They found that the reducing activity of plant extract was 335.8 ± 51.3, 31.4 ± 5.4, and 120.9 ± 12.6 µM TE when measured by ABTS, DPPH, and FRAR analyses, respectively. Moreover, the inhibitory activity for both Ag-and Au-NPs decreased by 60.7% and 44.7% for ABTS and 34.5% and 52.1% for FRAP compared to plant extracts, respectively. Both NPs also showed no significant change in DPPH activity compared to plant extract. The authors claimed that the reduction in the scavenging activity of NPs compared to plant extract was due to the consumption of plant biomolecules in the synthesis process of NPs. Ag- and Au-NPs synthesized using *Plumbago zeylanica* showed greater scavenging activity against DPPH with an inhibition percentage of 87.34% and 78.17%, respectively, compared to plant extract and an inhibition percentage of 71.16% and 74.88%, respectively, compared to standard butylated hydroxytoluene [85].

## 4. Conclusions

These findings demonstrated the potential use of *H. salicornicum* aqueous extract in synthesizing H@Ag-NPs and H@Au-NPs for the first time. GC-MS and FTIR data revealed that proteins, alcohol, and hydrocarbons (essential oils/terpenes) were the main phytochemicals in plant extract that might be responsible for reducing and stabilizing NPs. Our findings suggest that the variation in functional group compositions on the surface of NPs synthesized from the same plant may be due to responses to metal physicochemical characteristics, including the metal type, composition, and structure. The resultant H@Ag-NPs and H@Au-NPs had mainly uniform spherical shapes. H@Au-NPs also had some oval and triangular shapes. The size of H@Au-NPs (8.1 ± 0.3 nm) was smaller than that of H@Ag-NPs (19.1 ± 0.8 nm). In a colloidal system, H@Ag-NPs and H@Au-NPs have HD values of 184.7 nm, while H@Au-NPs had a HD of 56.4 and 295.4 nm. The surface of both NP types was negatively charged at about −24 mV. XRD, EDx, and mapping analyses indicated the crystallinity and the dominant distribution of silver and gold NPs in the samples. H@Ag-NPs showed great anticancer, antibacterial, and antioxidant activity compared to H@Au-NPs and plant extract. Although H@Au-NPs were smaller, their reactivity against cancer and microbial cells was weak, suggesting that the chemical properties, metal structure, quantity, and chemistry of the functional groups on the NP surface may influence their reactivity. Of the cancer cell types tested, Sw480 was the most sensitive to H@Ag-NPs, whereas Sw620 was the least permeable. These data indicated that the antiproliferative activity of H@Ag-NPs was cell response-dependent and may be affected by many factors, such as the cellular metabolic state that influences the cellular charge and interactions with charged NPs. Similarly, *S. aureus* (17.0 ± 0.12 mm) was the most sensitive bacteria to H@Ag-NPs, and *E. coli* (13.0 ± 0. mm) was the most resistant. The greater activity of H@Ag-NPs against Gram-positive vs. Gram-negative bacteria could be attributed to the nature of the NP charge, such that the negative charge of H@Ag-NPs allowed them to be electrostatically attracted to positively charged bacterial cell walls more than negatively charged bacterial cell walls. DPPH and FRAP assays showed that H@Ag-NPs exhibited greater scavenging and reducing activity against free radicals and FRAP reagents compared to plant extract and H@Au-NPs, suggesting that the higher reactivity and surface chemistry of silver ions may enable more plant-bioactive antioxidant molecules to bind on the NP surface to aid in increasing their antioxidant activity. The current data suggest that the use of *H. salicornicum* for the synthesis of Ag- and Au-NPs with a smaller size, uniform shape, and biological efficacy is an eco-benign, sustainable, fast, and effective method of NP production. Of note, the lower toxicity of H@Au-NPs enables these NPs to be used for drug delivery of traditional drugs. More investigations are required to understand the reactivity of NPs synthesized using *H. salicornicum* and the role of phytochemical functional groups on the NP surface to enhance the therapeutic activity of NPs. Performing intensive studies on the toxicological activity of H@Au-NPs may allow these NPs to be implemented for drug delivery at the clinical level.

## Figures and Tables

**Table 1:** The chemical compositions of *Haloxylon salicornicum* aqueous extracts analyzed by GC-MS.

No.	Compound	Retention Time	Area%	Matched Factor	Molecular Formula	Molecular Weight	Chemical Structure
1	1-Methyl-2-(1-methylethenyl)-4-(1-methylethylidene)-1-vinylcyclohexane), (1R-trans)-	10.12	0.64	846	C_15_H_24_	204	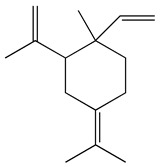
2	Cyclohexane, 1-ethenyl-1-methyl-2,4-bis(1-methylethenyl)-, (1R,2R,4S)-rel-	11.46	1.70	880	C_15_H_24_	204	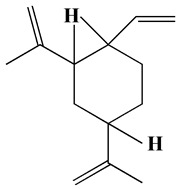
3	Aromandendrene	12.05, 12.45,12.87, 13.25, 13.45, 13.67, 14.76	0.80, 1.38, 0.38, 0.32, 0.70, 1.39, 0.71	873, 886, 893, 885, 856, 890, 868	C_15_H_24_	204	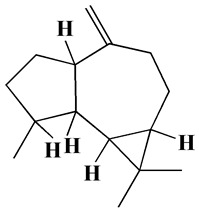
4	Beta-longipinene	13.16	0.46	828	C_15_H_24_	204	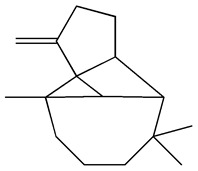
5	Eremophilene	13.88	1.65	902	C_15_H_24_	204	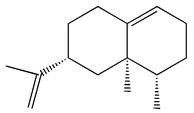
6	Alpha-copaene	14.03	1.30	788	C_15_H_24_	204	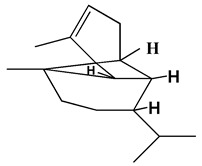
7	Gamma-himachalene	14.15	0.34	808	C_15_H_24_	204	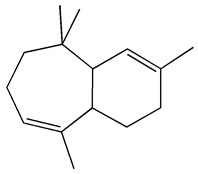
8	o-Menth-8-ene-4-methanol, α,α -dimethyl-1-vinyl-, (1S,2S,4R)-(-)-	14.43, 15.30	4.45, 3.18	899, 909	C_15_H_26_O	222	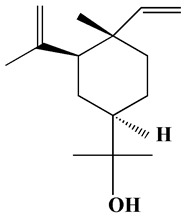
9	Beta-cadinene	14.56	2.55	807	C_15_H_24_	204	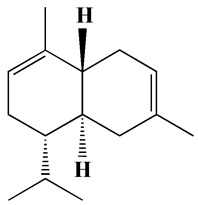
10	Beta-guaiene	14.92, 15.87	0.32, 0.31	765, 782	C_15_H_24_	204	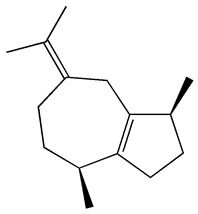
11	Murolan-3,9(11)-diene-10-peroxy	15.03	0.47	760	C_15_H_24_O_2_	236	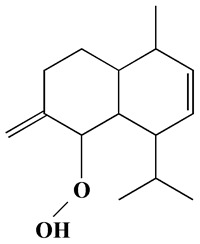
12	(-)-Globulol	15.13	0.48	829	C_15_H_26_O	222	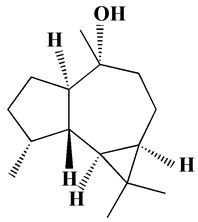
13	Alpha-farnesene	15.63	0.64	807	C_15_H_24_	204	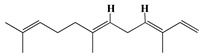
14	10-Epi-gamma-Eudesmol	16.20, 16.68, 17.10, 17.58	6.38, 6.90, 4.17, 6.24	870, 898, 865, 898	C_15_H_26_O	222	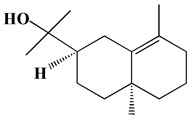
15	Guaiol	16.46	0.31	877	C_15_H_26_O	222	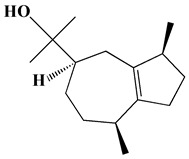
16	Eudesma-4(14),7(11)-diene	17.37	0.32	786	C_15_H_24_	204	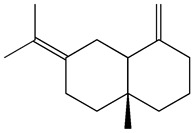
17	1-Chlorooctadecane	18.49, 20.56	1.31, 0.67	768, 755	C_18_H_37_Cl	288	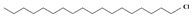
18	Methyl tetradecanoate	19.13	0.86	694	C_15_H_30_O_2_	242	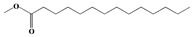
19	Cadalene	20.12	0.57	731	C_15_H_18_	198	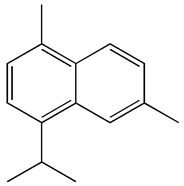
20	2-Acetyl-3-(2-cinnamido)ethyl-7-methoxyindole	20.68	0.52	615	C_22_H_22_N_2_O_3_	362	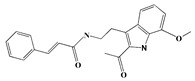
21	1,2-15,16-Diepoxyhexadecane	21.35	0.39	717	C_16_H_30_O_2_	254	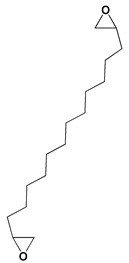
22	Phthalic acid, isobutyl octadecyl ester	22.07	0.52	828	C_30_H_50_O_4_	474	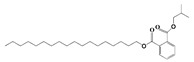
23	Ethyl linoleate	22.18	0.45	714	C_20_H_36_O_2_	308	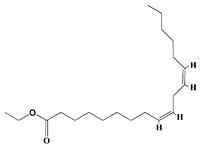
24	12,15-Octadecadiynoic acid, methyl Ester	22.72	0.49	713	C_19_H_30_O_2_	290	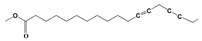
25	Methyl palmitate	23.22	10.50	906	C_17_H_34_O_2_	270	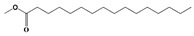
26	n-Hexadecanoic acid	24.59	3.96	831	C_16_H_32_O_2_	256	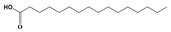
27	Cyclopropa[d]naphthalen-3-one, octahydro-2,4a,8,8-tetramethyl-, oxime	25.97, 26.77	1.87, 0.57	647, 675	C_15_H_25_NO	235	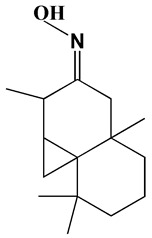
28	8,11-Octadecadienoic acid, methyl Ester	26.25	0.83	795	C_19_H_34_O_2_	294	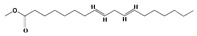
29	9-Octadecenoic acid (Z)-, methyl Ester	26.42, 26.53	6.81, 3.62	924, 885	C_19_H_36_O_2_	296	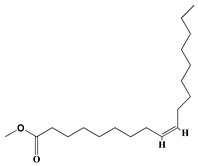
30	Methyl stearate	26.9	5.20	865	C_19_H_38_O_2_	298	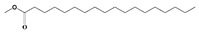
31	Methyl 6,8-octadecadiynoate	27.20	0.86	730	C_19_H_30_O_2_	290	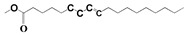
32	Oleic Acid	27.66, 27.99	3.04, 1.22	812, 774	C_18_H_34_O_2_	282	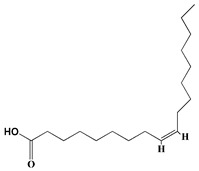
33	1,1-Dichloro-2-(2,2-dichloro-1-methylcyclopropyl)-2-methylcyclopropane	28.83	4.97	687	C_8_H_10_Cl_4_	246	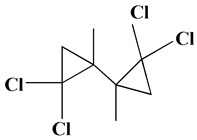
34	Methyl 14-methyl-eicosanoate	30.25	0.65	701	C_22_H_44_O_2_	340	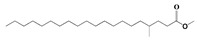
35	Dodecanoic acid, 10-methyl-, methyl Ester	33.38	0.69	771	C_14_H_28_O_2_	228	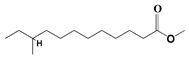
36	Phthalic acid, 5-methylhex-2-yl heptadecyl ester	33.67	0.93	805	C_32_H_54_O_4_	502	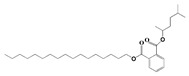

**Table 2:** FTIR spectra of *Haloxylon salicornicum* extract, H@Ag-NPs and H@Au-NPs and its corresponding functional groups.

FTIR Spectra (cm^−1^)	Functional Gtoups of *Haloxylon salicornicum*	FTIR Spectra (cm^−1^)	Functional Gtoups of H@Ag-NPs	FTIR Spectra (cm^−1^)	Functional Gtoups of H@Au-NPs
3368.04	O-H	3424.7	O-H	3424.7	O-H
1634.5	N-H	2928.8	C-H	2928.8	C-H
1467.1	C-H	2861.8	C-H	2861.8	C-H
1409.01	O-H	1634.0	C=C	1747.4	C=O
1330.7	C-N	1510.3	N-O	1634.0	C=C
1251.4	C-O	1386.5	C-H	1465.9	C-H
1093.8	C-O	1240.2	C-N	1231.0	C-N
907.4	C=C	1059.7	C-O	1173.2	C-O
829.9	C-Cl	587.6	C-Cl	1059.7	C-O
721.2	C=C			582.0	C-Cl
609.7	C-Cl				
500.0	C-I				

**Table 3:** Elemental compositions of both H@Ag-NPs and H@Au-NPs analyzed by energy diffraction X-ray.

H@Ag-NPs				H@Au-NPs			
Elements	Line	Mass%	Atom%	Elements	Line	Mass%	Atom%
C	K	2.55 ± 0.01	16.44 ± 0.06	C	K	3.97 ± 0.02	35.88 ± 0.14
O	K	0.58 ± 0.01	2.80 ± 0.06	Al	K	0.21 ± 0.01	0.85 ± 0.04
Al	K	0.14 ± 0.01	0.41± 0.02	Cu	K	5.54 ± 0.08	9.45 ± 0.13
Cl	K	7.08 ± 0.02	15.46 ± 0.05	Zn	K	3.73 ± 0.09	6.18 ± 0.15
Cu	K	1.04 ± 0.04	1.26 ± 0.05	Au	M	86.55 ± 0.14	47.64 ± 0.08
Ag	L	88.61 ± 0.10	63.62 ± 0.07				
Total		100	100	Total		100	100

**Table 4:** Minimum inhibition and bactericidal concentrations (MIC and MBC) of H@Ag-NPs and H@Ag-NPs and *H. salicornicum* aqueous extract against *Staphylococcus aureus*, *Bacillus cereus*, *Escherichia coli*, and *Klebsiella pneumoniae*.

Bacteria	H@Ag-NPs (µg/mL)		H@Au-NPs (µg/mL)		*H. salicornicum* (µg/mL)	
	MIC	MBC	MIC	MBC	MIC	MBC
*S.aureus*	1.95	3.9	>500	>500	>500	>500
*B. cereus*	3.90	7.8	>500	>500	>500	>500
*K. pneumoniae*	3.90	7.81	>500	>500	>500	>500
*E. coli*	7.8	15.6	>500	>500	>500	>500

**Table 5:** Inhibition zone diameter (mm) of H@Ag-NPs and H@Ag-NPs and *H. salicornicum* aqueous extract against *Staphylococcus aureus*, *Bacillus cereus*, *Escherichia coli*, and *Klebsiella pneumoniae*.

Strains	Ciprofloxacin	H@Ag-NPs	H@Au-NPs	*H. salicornicum*
*S.aureus*	30.0 ± 0.2	17.0 ± 0.1	0.0 ± 0.0	12.0 ± 0.1
*B. cereus*	32.0 ± 0.3	13.4 ± 0.2	0.0 ± 0.0	11.0 ± 0.2
*E. coli*	30.0 ± 0.2	13.0 ± 0.3	0.0 ± 0.0	9.8 ± 0.3
*K. pneumoniae*	32.0 ± 0.2	13.3 ± 0.4	0.0 ± 0.0	9.9 ± 0.6

## Data Availability

Additional data to those presented here are available from the corresponding author upon reasonable request.
